# Project Gender NutriScope: Methods of a Mixed Methods, Community-Engaged Study Design to Explore the Nutritional Needs of Transgender and Gender-Diverse Youth and Young Adults

**DOI:** 10.1016/j.jneb.2025.01.001

**Published:** 2025-02-06

**Authors:** Heather E. Schier, Krithika Chetty, Shivakriti Induri, Liam Gallagher, Miriam Knopp, Julie Kennel, Whitney Linsenmeyer, Avery M. Anderson, Matthew Adkins, Irene E. Hatsu, Janna D. Stephens, Carolyn Gunther

**Affiliations:** 1 Department of Nutrition and Healthcare Management, Appalachian State University, Boone, NC; 2 Department of Human Sciences, The Ohio State University, Columbus, OH; 3 Department of Public Health, The Ohio State University, Columbus, OH; 4 College of Arts and Sciences, The Ohio State University, Columbus, OH; 5 Kaleidoscope Youth Center, Columbus, OH; 6 Nationwide Children’s Hospital, Columbus, OH; 7 Department of Nutrition and Dietetics, Saint Louis University, St. Louis, MO; 8 College of Nursing, University of Colorado Anschutz Medical Campus, Aurora, CO; 9 Grant Family Medicine Residency, OhioHealth, Columbus, OH; 10 Department of Population Health Nursing Science, College of Nursing, University of Illinois-Chicago, Chicago, IL; 11 Graduate Studies Committee, College of Nursing, Columbus, OH

**Keywords:** gender identity, transgender persons, nutrition assessment, exploratory methods, community-engaged

## Abstract

**Objective:**

Despite growing evidence of distinct nutrition-related experiences and disparities, transgender and gender-diverse (TGD) youth and young adults are an underrepresented population in nutrition research. This paper describes the methods and study design from Project Gender NutriScope, a study that will explore the nutritional needs of TGD youth and young adults.

**Design:**

Parallel convergent cross-sectional mixed methods; community-engaged.

**Setting:**

A purposive sample will be recruited in a Midwest city through clinics, youth organizations, and a large state university.

**Participants:**

Transgender and gender-diverse youth and young adults aged 13–24 years.

**Intervention:**

Findings from this formative study will be used to inform future intervention development.

**Main Outcome Measures:**

Dietary intake, eating patterns, disordered eating patterns, food security status, perceptions of relationship with food, and nutrition-related concerns.

**Analysis:**

Quantitative data will be analyzed using descriptive summary statistics. Qualitative data will be analyzed by reflexive thematic analysis. The 2 databases will be integrated iteratively.

## INTRODUCTION

Nutrition-related clinical and psychosocial implications are associated with diverse gender experiences.^1–3^ For example, transgender and gender-diverse (TGD) individuals who undergo gender-affirming hormone therapy (GAHT) can experience adaptations to body composition and altered appetite.^4,5^ Gender-based discrimination can exacerbate nutritional health disparities (e.g., food insecurity, disordered eating).^6–8^ Research indicates that TGD individuals experience significantly higher rates of eating disorders compared with cisgender individuals, with studies showing prevalence rates as high as 15% to 35% in some TGD populations, which is substantially higher than the general population average of 1% to 4%.^6,8–10^ Despite growing evidence of distinct nutrition-related experiences and disparities, TGD youth and young adults are an underrepresented population in nutrition research.^2,11^ The need for tailored health services across the gender continuum is increasing, including a demand for nutrition care services.^1,2,12^

Sometimes described as hard-toreach or hidden populations, underrepresented groups face distinct challenges to participating in research that is underpinned by oppressive and discriminatory social fabrics.^13^ Researchers must consider effective evidence-based and justice-informed strategies to connect and work with underrepresented groups.^14^ This protocol paper describes the methods and study design of Project Gender NutriScope, a cross-sectional, community-based, parallel convergent mixed-methods study to investigate the nutritional concerns of TGD youth and young adults. This study aimed to characterize the nutritionrelated behaviors and concerns of TGD youth and young adults (aged 13–24 years) residing in the Columbus, Ohio, metropolitan area.

The specific aims are to (1) describe the nutritional needs of TGD youth and young adults using cross-sectional surveys and direct measure data and (2) explore the food and nutrition concerns of TGD youth and young adults through indepth interviews.

Results from this hypothesis-generating study will inform future recruitment methods and community-centered research questions and ultimately contribute to the development of evidence-based nutrition care practices, interventions, and guidelines for TGD youth and young adults.

## METHODS

### Theoretical Frameworks

Project Gender NutriScope is firmly rooted in a transformative world-view with a constructivist perspective, emphasizing the dynamic and socially constructed nature of reality. This perspective seeks to challenge oppressive systems and promote social justice, when knowledge is viewed as a subjective creation collaboratively shaped by researchers and participants.^15^ This approach places a strong emphasis on the active construction of meaning through interpretation, valuing diverse perspectives, and considering the contextual nuances that inform the research questions, design, and interpretations within the framework of mixed-methods research.^15^

In addition to this foundational perspective, our study will use principles of the Health Equity Promotion Model.^16^ Affirming the right to health and wellness for all individuals, this model provides a comprehensive framework for understanding how multilevel factors intersect to influence health outcomes throughout the lifespan, particularly within lesbian, gay, bisexual, transgender, and queer (LGBTQ+) populations.^16^ The model takes into account both the pathways that promote health (e.g., resilience, agency, responses to adversity) and adverse determinants (e.g., discrimination, societal structures, stigma) along the health continuum.^16^ Importantly, it acknowledges the rich tapestry of experiences within and across subpopulations, considering factors such as race, ethnicity, sexual orientation, gender identity, nativity, disability status, socioeconomic status, and more. Furthermore, it adopts a life course perspective, recognizing the influence of time, historical context, cultural factors, and life course positioning on experiences, coping strategies, and outcomes.^16^

**Figure 1.**
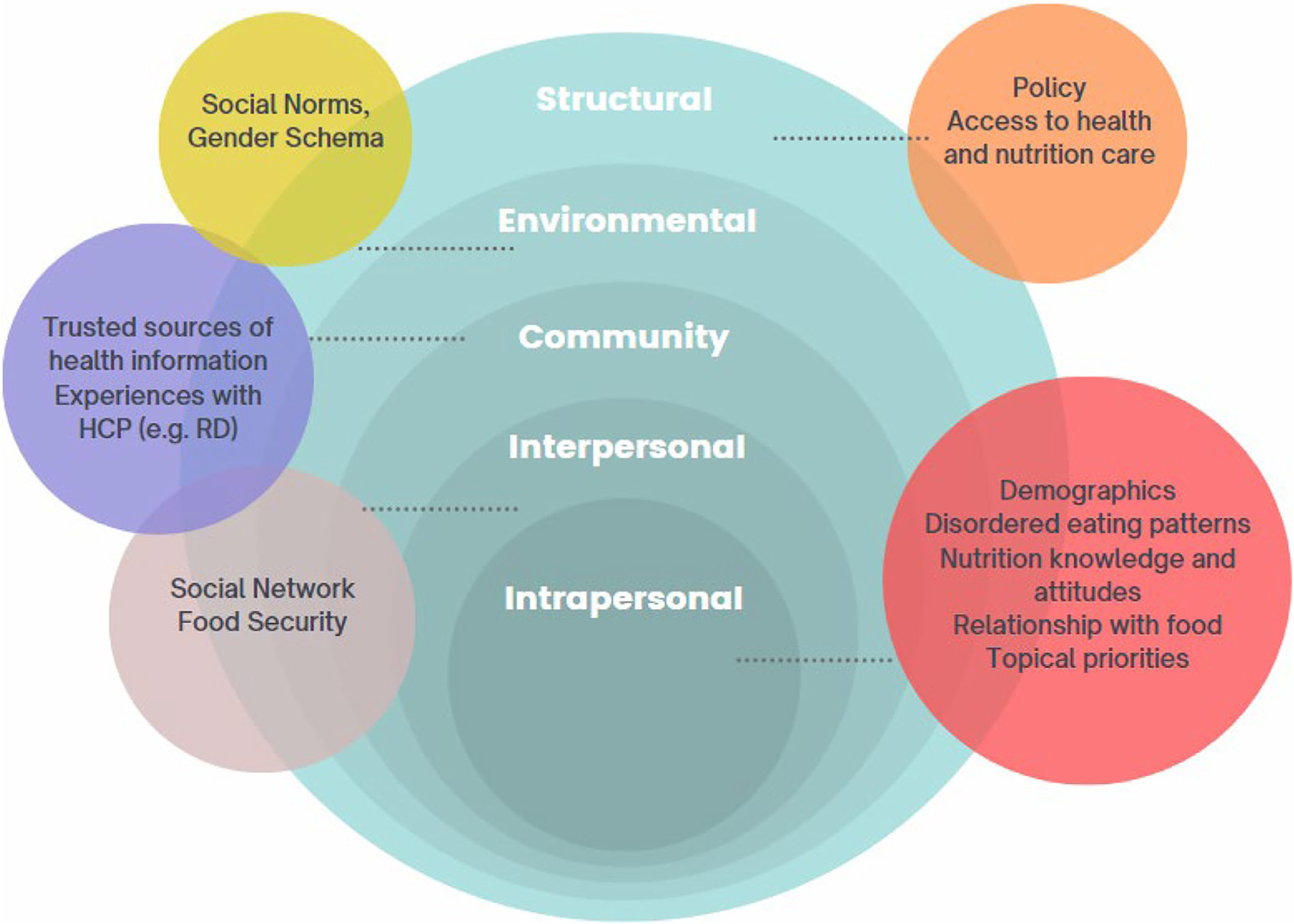
The integrated multilevel theoretical framework underpinning the study design for Gender NutriScope. HCP indicates health care professional; RD, registered dietitian.

Complementing these perspectives, Bronfenbrenner’s socioecological model will also have a prominent influence on the study design.^17^ This model provides a lens through which to understand the intricate interplay among factors across 4 social levels: intrapersonal, interpersonal/household, community, and institutional/policy.^17^ Figure 1 outlines the integration of elements from both the Health Equity Promotion Model and the socioecological model. This combined framework will serve as the bedrock for every step of the research process.

Finally, although identifying disparities affecting gender minority groups is paramount to addressing health equity, a deficit-focused model fails to empower participants and build community resilience. Therefore, in addition to illuminating and examining health disparities, this work aims to celebrate and empower diverse gender identities.

### Study Design

**Figure 2.**
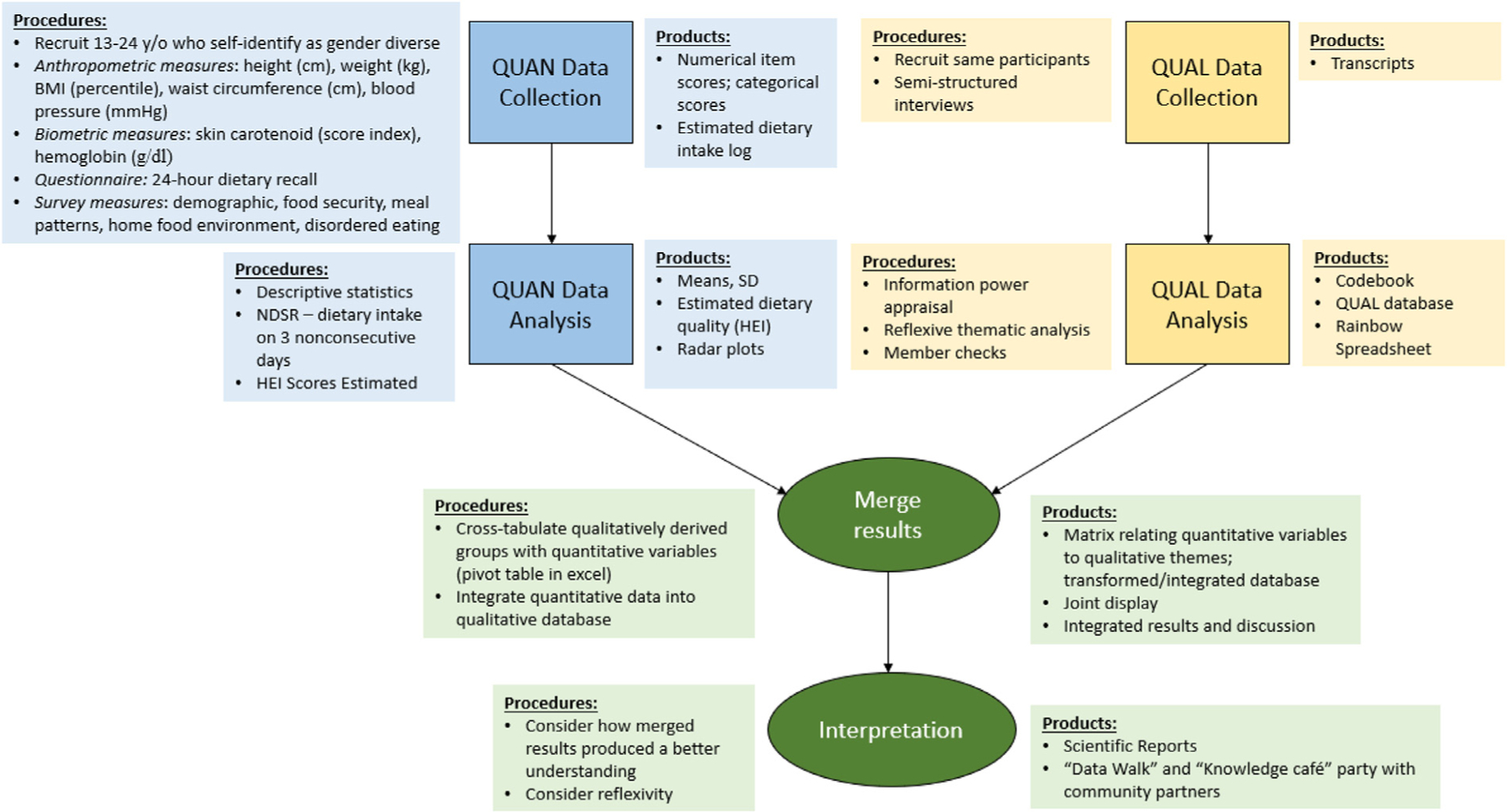
Diagram of the parallel convergent mixed-methods study design for Gender NutriScope. BMI indicates body mass index; HEI, Healthy Eating Index; NDSR, Nutrition Data System for Research; QUAL, qualitative; QUAN, quantitative; y/o, years old.

This cross-sectional study will employ a convergent parallel mixedmethods design through a community-based research approach (Figure 2). Quantitative and qualitative data will be collected concurrently, analyzed separately, and then integrated.^15^ All study materials and procedures have undergone expedited review and have been approved by The Ohio State University Institutional Review Board (2021B0338).

### Research Team

#### Community advisory board.

The community advisory board (CAB) will consist of community members and experts in various relevant fields. The collective group will possess extensive experience in TGD youth outreach and TGD health care, including gender-affirming care, nutrition, and mental health services. All members will play an integral role as equitable contributors to the research team by participating in the review and feedback process for all recruitment materials, survey tools, and interview guides. Importantly, the CAB will meet to co-analyze and interrogate the findings alongside the research team, offering depth to the interpretations. The CAB will be scheduled to convene regularly with the research team on a quarterly basis and maintain ongoing collaborative communication via email as needed. One community partner will engage biweekly with the research team to provide consistent input and guidance. This comprehensive engagement will ensure the depth and rigor of the research findings’ interpretation. Community advisory board members will not be compensated for their contributions, but authorship will be offered as a benefit to those who agree to contribute to manuscript preparation.

#### Establishing community partnerships.

Community partners will be established over a year of building relationships with organizations and health care providers. The primary research team will do so through regular meetings, attending partners’ events, and sharing why the project is important to the research team. For example, the primary research team will meet with staff and youth during regular community organization programming to discuss the importance of the project and build rapport, as it can take time to build trust with lesbian, gay, bisexual, transgender, queer/questioning, intersex, and asexual/aromantic/agender youth. Meeting with the youth in an informal setting will ensure the research team is able to build a foundation of trust with the youth participants before recruitment.

### Participants and Setting

A purposive sample will be recruited on a rolling basis until information power is satisfactory.^16^ Information power refers to the extent to which the data collected from participants can contribute to answering the research questions, achieving the study’s aims, and providing deep insights into the phenomena being studied. It emphasizes the value of having participants who can offer substantial, meaningful, and nuanced information, which can lead to more comprehensive and impactful research findings.^18^ Eligibility criteria will include being aged 13–24 years, self-identifying as gender diverse, and residing in Columbus, Ohio. Recruitment sites will include community-based organizations and will involve in-person engagement with participants during community events, distribution of flyers at recruitment sites, and online engagement through social media and websites. This recruitment approach was selected based on its known effectiveness in reaching diverse gender-marginalized individuals.^19^ The target enrollment of 10 participants was determined based on information power estimations.^18^ Participants will receive a $45 Visa gift card after completing the study.

### Data Collection

The research team will complete comprehensive training in gender-affirming engagement and data collection protocols, including competency assessments like blood pressure checks. Parental permission and participant assent (aged 13–17 years) or consent (aged 18–24 years) will be obtained. Validated survey and semistructured interview guides will be internally pilot-tested with CAB members, including the target population and topic experts.^20^ Revisions will be made in response to key informant input. The data collection sessions (estimated 60–75 minutes) will include electronic surveys, an interview, and physical measurements. Dietary recall data will be collected on 3 nonconsecutive days, the first during the study appointment and the remaining 2 during unannounced telephone calls.^21^ Physical measurements (Figure 2) will be collected in person by trained research staff in a private space. Participants will have the option to complete surveys and interviews virtually, but physical measurements will require an in-person appointment.

One-on-one semistructured interviews were selected because of the personal and sensitive nature of the interview questions and the desire to respect the privacy of individuals.^22^ All interviews will be audio recorded and transcribed verbatim using a naturalist approach.^23^ In this approach, the conversation will be captured exactly as it occurs. The meaning, all hesitations, interjections, stutters, and so on will be included.^23^ Audio files will be first transcribed using the dictate function on Microsoft Word. The researchers will then listen to the audio file and correct the dictation results as needed. During the first pass or familiarization phase, the lead researcher will add clarifying comments and mark unclear or missing audio with ellipses.^24^

### Outcome Measures

Multilevel theoretical frameworks^16,17^ guided the selection of the outcome measures (Table 1). The survey modules explore the relationship between gender identity and nutritional concerns and needs through the following constructs: intrapersonal (personal dietary behaviors and patterns, access to food) and interpersonal/household (socioeconomic status, home food environment). Validated surveys with minor adaptations based on key informant feedback and target population considerations (i.e., age and gender identity) will be used. The surveys include: a demographic survey, the US Department of Agriculture’s (USDA) multiple-pass 24-hour dietary recall,^25,26^ Adolescent Meal Consumption Patterns Survey,^27^ Family Mealtime Survey,^28,29^ the Eating Disorder Examination Questionnaire-Adolescents (EDE-A),^30,31^ and the USDA Child Food Security 9-item Survey.^32^ The demographic survey will be constructed using questions from the broad literature and published recommendations on how to best assess gender and sex using the 2-step method.^11,33^ Gender-affirming medical interventions (e.g., GAHT, surgery, etc.) will be captured. Participants’ socioeconomic status will be measured using questions from the home affluence score^34,35^ rather than inquiring about caregiver employment or income, which are historically difficult questions for adolescents to answer. The home affluence score will be scored on a scale of 0–4, with higher scores estimating a higher socioeconomic status.^34^ Diversity in prompt and response formatting (e.g., a combination of multiple choice, scales, and open-ended questions) was selected to prevent survey fatigue.

#### Diet quality.

The USDA’s 5-step multiple-pass 24-hour dietary recall method will be used to estimate dietary intake and calculate Healthy Eating Index scores.^25,26^ This is the preferred method of diet assessment when comparing intake to recommendations, like the Dietary Guidelines for Americans,^36^ and calculating mean values for a group.^37^ Furthermore, the openended nature of a recall lends to capturing information about specific diet choices and eating patterns.^37^ Diet intake data for each participant will be entered into the Nutrition Data System for Research (version 2022) to analyze Healthy Eating Index scores as an estimation of participant diet quality.^38^

#### Eating and meal patterns.

The adolescent meal consumption patterns and family mealtime surveys will be administered to capture the frequency of skipping meals and elements of the home food environment (e.g., family meal frequency, electronic device use during meals).^27–29^ The adolescent meal consumption patterns survey focuses on how often individuals eat meals, which meal they eat most often, and how often they replace meals with snacks. The family mealtime survey measures how often meals are eaten with family over the past 7 days, the location of mealtime, whether participants are involved in meal preparation if take-out meals were purchased, and whether digital devices (e.g., television, tablets, smartphones, etc.) were present during meals.

The EDE-A is a 36-item self-report measure designed and validated to assess eating disorder symptoms and attitudes in adolescents over the past 14 days.^30,31^ Community norms have been established using the Eating Disorder Examination Questionnaire (EDE-Q) for the transgender adult population.^39^ Therefore, the EDE-A, an adaptation of the EDE-Q for adolescents,^30,31^ was selected as the best fit over other eating disorder pattern modules. Responses will yield a global score, which will be calculated by averaging the scores from 4 subscales: (1) restraint, (2) eating concern, (3) weight concern, and (4) shape concern.^30,31^

#### Household food security.

US Department of Agriculture’s Child Food Security 9-Item Survey is a widely used validated tool to assess food security in households with children.^32^ Child Food Security 9-Item Survey scores are categorized into the following levels of food security: high, marginal, low, and very low food security.^32^

#### Anthropometric and biometric protocols.

Anthropometric data (nutritional status/general health), blood pressure (cardiometabolic health), skin carotenoid (fruit and vegetable intake biomarker), and hemoglobin (iron status biomarker) will be collected using the National Health and Nutrition Examination Survey protocol.^40^ For participants aged < 20 years, body mass index (BMI) percentiles will be used to assess growth and body composition relative to age and sex-specific reference values.^41^ For participants aged 21–24 years, actual BMI measurements will be used to evaluate body composition, as percentile-based assessments are not applicable for this older age group. Growth charts consistent with the sex assigned at birth for participants who have not begun GAHT will be used to determine BMI. For participants who indicate initiation of GAHT, BMI will be reported as a range using the growth chart for both sexes.^42^ Skin carotenoid levels, a noninvasive measure of skin concentrations of carotenoids, will be measured using Resonance Raman light scattering spectroscopy.^37,43^ Finger-prick point-of-care testing of hemoglobin will be conducted with a Hemocue analyzer to assess iron status.^44^ Hemoglobin was chosen over serum ferritin as a biomarker for iron status in this study because of its practicality and relevance. Hemoglobin is widely used to assess iron status and severe anemia in both clinical and field settings, providing immediate and actionable information.^45^ It is also more cost-effective and logistically simpler to measure than serum ferritin, requiring only a small blood sample and portable equipment, which is ideal for a study executed within community spaces. In addition, hemoglobin levels are directly affected by GAHT, particularly testosterone, which can increase hemoglobin and hematocrit levels.^46^ This makes hemoglobin especially relevant for monitoring iron status in TGD youth and young adults. Furthermore, hemoglobin reflects more immediate changes in iron status and is less affected by inflammation than serum ferritin,^47^ offering clearer insights into the participants’ current iron status. Overall, hemoglobin provides a practical and straightforward measure for understanding the iron status of our study population.

#### In-depth interviews.

The accompanying original interview guide (Table 2) will be refined in collaboration with the CAB to elicit the concerns and needs germane to nutritional health among TGD youth and young adults. The interview guides will be constructed a) based on key constructs to characterize fundamental nutrition concerns and needs and b) as a complement to the quantitative survey modules. The constructs, derived from the Health Equity Promotion Framework and socioecological model (Figure 1), are outlined in Table 2 and include nutrition education and existing knowledge; relationship with nutrition, diet, and food; target population nutrition, diet, and food priorities; current medical transitioning status; social support; experience with health professionals; and trusted resources. The interviews will be conducted by a trained researcher from the LGBTQ+ community with extensive experience in qualitative research, ensuring both high-quality data collection and sensitivity to the participants’ perspectives through rigorous training and personal understanding. A second research assistant will be present to transcribe the interviews and take notes, ensuring comprehensive documentation and support during the data collection process.

### Data Analysis

Quantitative and qualitative data will be collected concurrently, analyzed separately, and then integrated (Figure 2).^15^ Quantitative data will be analyzed using descriptive statistics. Qualitative data will be analyzed using reflexive thematic analysis (RTA), which is a qualitative research method that systematically organizes and categorizes data, followed by a reflexive analysis to identify and report patterns or themes.^48^ It involves critically examining the researcher team’s role in shaping data analysis and interpretation. It is particularly valuable for open-ended exploratory studies, like this one conducted by researchers outside the target community.^48^ Reflexive thematic analysis will be conducted through 6 iterative steps by 3 coders: data familiarization, initial coding, theme search, theme review, theme naming and defining, and the final report.^48^

Data familiarization will entail a comprehensive review of transcripts during the transcription process and will be performed independently by each coder. Initial coding, assignment of concepts or terms to text, will be manually conducted using *ad hoc* coding of segments based on their conceptual significance and permitting multiple codes for each segment. Coders will convene to discuss and enhance the interpretation of data segments, ensuring complexity and nuance in code assignments. Subsequently, initial codes and subcodes (related concepts or terms nestled under initial codes) will be organized into a coding frame guided by the selected theoretical frameworks. Theme development and refinement will involve sorting coded data to discern meaningful trends. Themes will be subjected to iterative review and refinement by the research team, CAB, and participants (member checks) to enhance data interpretation.

### Reflexivity Statement

Outsiders, those who are not part of the targeted community, offer a unique perspective that can reveal insights missed by insiders. Their fresh viewpoint and willingness to ask naive questions can lead to in-depth exploration and valuable data.^49–52^ However, this outsider perspective, particularly when studying marginalized populations, demands careful ethical considerations. Critics express concerns about outsiders comprehending and accurately representing participant experiences.^49^ Addressing this issue involves bridging the psychological and social gap between the researcher and participants, demonstrating cultural sensitivity, and contributing positively to both participants’ lives and broader understanding.^53,54^ Our primary research team, consisting of cisgender females (one of whom is queer) committed to transparency and reflexivity, will collaborate closely with transgender members of our CAB, who will play a pivotal role in shaping research questions, methodology, and interpretation of findings. We will remain dedicated to conducting respectful and communitycentered research.

## DISCUSSION

In recent years, there has been a 2-fold increase in the number of TGD individuals in the US.^55^ Achieving gender affirmation often involves legal, social, and medical considerations as well as associated clinical and psychosocial implications.^2^ However, despite evidence suggesting that TGD youth and young adults face nutrition-related challenges, limited evidence-based nutrition care practices, interventions, or guidelines exist.^2^ An emerging body of research highlights unique nutrition concerns among TGD youth and young adults,^4,6,10,12^ but quantitative or qualitative data describing their dietary patterns, nutritional health, and broader perspectives on food and nutrition are scarce.^2^ This research gap hampers the ability of nutrition professionals to provide evidence-based, affirming care for TGD youth and young adults, potentially exacer-bating health disparities within this marginalized population. To address this gap, data supports researchers considering effective implementation strategies such as investing in meaningful community partnerships and leveraging equity-centric theoretical frameworks.

The following 3 paragraphs describe methodological critiques and alternative approaches. Describing the limitations and critiques of the methods selected promotes transparency, is critical for the applicability of findings, and enhances future research designs.

Purposive sampling was selected because of its feasibility and appropriateness for exploratory and qualitative research. However, there are limitations, including the inability to measure systematic error and limitations in the generalizability of findings.^56^ When conducting community nutrition research, it can be difficult to reach marginalized populations. This can result in unrep-resentative data when using non-probability-based or convenience sampling methods.

The researchers will use 2 methods of discerning an appropriate participant group size: information power^18^ and data saturation. The data saturation method is designed for thematic analyses; saturation will be calculated as interviews are conducted and analyzed, reflecting the real-time thresh-old at which no new information is learned.^57^ Reflexive thematic analysis research experts Braun and Clark^58^ argue that calculating data saturation *a priori* is aligned with grounded theory but misaligned with RTA—the approach being employed for this study. Researchers using RTA are encouraged to embrace uncertainty and acknowledge that meaning is constructed through the interpretation of data rather than discovery. Therefore, decisions about how much data to collect and when to stop data collection are subjective and context-dependent and cannot be predetermined with certainty. It is less clear how calculating saturation throughout the analysis process, as described by Guest et al.,^57^ fits in with this argument. However, it’s worth noting that regardless of the timing of calculating data saturation, doing so may hold little value because of the subjective nature of RTA. To account for this, both data saturation and information power will be used to discern the appropriateness of the participant group size.

The limitations of self-reported diet assessments are well documented.^37,59^ While the 24-hour recall offers several strengths, there are limitations to the accuracy of the diet estimations. For example, the number of days (typically 1–3 days) does not represent usual intake,^37,60^ accurately expressing portion sizes is difficult,^61^ participant memory lapses,^37^ biases are common (e.g., underestimation of food perceived as unhealthy)^62^ and nutrient values of foods vary across nutrient composition databases.^37^ Using the Automated Self-Administered 24-Hour Dietary Assessment tool may yield more accurate results because of the visual representations that help estimate portions and stimulate memory.^63^ Though pretraining participants on the use of food models to estimate portions have been shown to improve the accuracy of recall, this approach was not selected for this protocol.^64^

In this study, the methods of rigor include convening a CAB, conducting pilot tests and member checks with the community, and iteratively reflecting on the impact of the researchers’ identities, experiences, and positionality across all research components. Triangulation will be achieved through the use of multiple data types and 2 theoretical frameworks. Extensive reflexivity will play a crucial role in ensuring transparency in the generation of knowledge.^24,48^ It is worth noting that intercoder agreement and data saturation, both derived from a positivist orientation, will be calculated despite misalignment with the study’s orientation to satisfy the positivist view of many nutrition science researchers and reviewers. Braun and Clark,^24^ among other scholars, have discussed limitations associated with calculating intercoder agreement and data saturation in RTA.^48^ Furthermore, the collaborative research team will discuss anticipated challenges (e.g., recruitment of a sufficient and diverse participant group) and identify ways to adapt approaches to both uphold the study’s integrity and accommodate challenges. For example, offering flexibilities to enhance accessibility to participate and considering alternative recruitment outlets.

This paper describes the rigorous methods of Project Gender Nutri-Scope, the first community-based, parallel convergent mixed-methods study underpinned by theoretical frameworks (Health Equity Promotion Framework and the socioecological model) to characterize nutrition-related concerns of TGD youth and young adults. As the urgency for TGD-focused nutritional clinical services and research grows, this work must be grounded in community engagement. Findings from Project Gender NutriScope will be disseminated in forthcoming articles and used to inform future research and intervention development.

## Figures and Tables

**Table 1.** Application of the Socioecological Model in the Gender NutriScope Project: Theoretical Framework, Data Collection, and Methods

Ecological Level	Data Collected	Method
Individual	Demographics Anthropometrics Biometrics Dietary intake Eating and meal patterns Nutrition education and knowledge Relationship with food Topical priorities	Measurement 24-hour dietary recall questionnaire Survey In-depth interviews
Interpersonal	Demographics Anthropometrics Biometrics Food security Eating and meal patterns Social support Experience with health care professionals	Measurement Survey In-depth interviews
Community	Food security Trusted resources Experience with health care professionals Nutrition education and knowledge	In-depth interviews
Structural	Food security Experience with health care professionals	In-depth interviews

**Table 2.** Alignment of Interview Questions with Theory-Based Constructs and Research Objectives in Exploring Nutrition-Related Experiences of Transgender and Gender-Diverse Youth and Young Adults

Interview Question	Construct/Objective
Please tell me about your interest in nutrition and food	Build rapport, ease into the topic
Has anyone ever spoken to you about nutrition, diet, or food consumption?	Nutrition education and existing knowledge
If yes, PROBE (Who, when, what was discussed?)	
How do you feel when you talk about nutrition, diet, or food consumption?	Relationship with nutrition, diet, food
How would you describe your relationship with food?	Relationship with nutrition, diet, food
Has anything changed [with your relationship with food] over the last X years?	Relationship with nutrition, diet, food
If yes, PROBE (What do you think has led to those changes?)	
If you were to have an appointment with a registered dietitian or nutritionist, what nutrition, diet, or food-related topics would you like to learn more about?	Target population nutrition/food priority topics
What are your biggest health concerns for yourself? For others? For other trans peers?	Target population nutrition/food priority topics
What are your biggest nutrition-related concerns for yourself?	Target population nutrition/food priority topics
Medical transitioning can affect your body—therefore we’re interested in what medical transitioning services you’ve received so far. Have you undergone any medical transitioning steps?	Identify current medical transitioning status
If needed, PROBE (e.g., puberty blockers, hormone therapy, or any surgeries)	
If yes, are you comfortable telling me a little bit about which steps you’ve taken?	Identify current medical transitioning status
PROBE: Where did you learn about your medical options? Who has supported you through those steps? Did those providing medical advice discuss any nutrition-related topics with you either before, during, or after care?	Identify current medical transitioning status, social support, experience with health professionals, trusted resources
In what ways have you or someone you know used food or beverages to support transitioning?	Target population nutrition/food priority topics
What sources have you or your trans peers used to seek nutrition-related information?	Trusted resources
PROBE: YouTube, Reddit, TikTok, friends/peers, support groups, medical professionals, etc.?	
If you or a trans peer were to seek nutrition-related information on the internet, what types of questions might you search?	Target population nutrition/food priority topics
What websites might you use to conduct your search?	Trusted resources; target population nutrition/food priority topics
Who are the most trustworthy people you feel comfortable receiving health related information from?	Trusted resources
PROBE: Friends/peers, community organization leaders, medical professionals, mentors, etc.?	
RESOURCES (curated by the community advisory board)	LGBTQ+-focused services in the areas of mental health, disordered eating, food security, and general health and well-being

LGBTQ+ indicates lesbian, gay, bisexual, transgender, intersex, queer/questioning, asexual, and many other terms (such as nonbinary and pansexual).

## References

[1] Linsenmeyer W Sex and gender in nutrition research: considerations for transgender and gender nonconforming populations. Proc Nutr Soc. 2024;83: 76–81. 37731252 10.1017/S0029665123003683

[2] Rozga M, Linsenmeyer W, Cantwell Wood J, Darst V, Gradwell EK. Hormone therapy, health outcomes and the role of nutrition in transgender individuals: a scoping review. Clin Nutr ESPEN. 2020;40:42–56. 33183572 10.1016/j.clnesp.2020.08.011

[3] Rahman R, Linsenmeyer WR. Caring for transgender patients and clients: nutrition-related clinical and psychosocial considerations. J Acad Nutr Diet. 2019;119:727–732. 29779913 10.1016/j.jand.2018.03.006

[4] Linsenmeyer W, Rahman R, Stewart DB. The evolution of a transgender male’s relationship with food and exercise: a narrative inquiry. J Creat Ment Health. 2022;17:2–14.

[5] Linsenmeyer W, Drallmeier T, Thomure M. Towards gender-affirming nutrition assessment: a case series of adult transgender men with distinct nutrition considerations. Nutr J. 2020;19:74. 32677957 10.1186/s12937-020-00590-4 PMC7367386

[6] Linsenmeyer WR, Katz IM, Reed JL, Giedinghagen AM, Lewis CB, Garwood SK. Disordered eating, food insecurity, and weight status among transgender and gender nonbinary youth and young adults: a cross-sectional study using a nutrition screening protocol. LGBT Health. 2021;8:359–366. 34097472 10.1089/lgbt.2020.0308

[7] Tan KKH, Treharne GJ, Ellis SJ, Schmidt JM, Veale JF. Gender minority stress: a critical review. J Homosex. 2020;67:1471–1489. 30912709 10.1080/00918369.2019.1591789

[8] Arikawa AY, Ross J, Wright L, Elmore M, Gonzalez AM, Wallace TC. Results of an online survey about food insecurity and eating disorder behaviors administered to a volunteer sample of self-described LGBTQ+ young adults aged 18 to 35 years. J Acad Nutr Diet. 2021;121:1231–1241. 33158800 10.1016/j.jand.2020.09.032

[9] Avila JT, Golden NH, Aye T. Eating disorder screening in transgender youth. J Adolesc Health. 2019;65:815–817. 31500946 10.1016/j.jadohealth.2019.06.011

[10] Ferrero EM, Yunker AG, Cuffe S, Nutrition and health in the lesbian, gay, bisexual, transgender, queer/questioning community: a narrative review. Adv Nutr. 2023;14:1297–1306. 37536566 10.1016/j.advnut.2023.07.009 PMC10721458

[11] Schier HE, Gunther C, Landry MJ, Ohlhorst SD, Linsenmeyer W. Sex and gender data collection in nutrition research: considerations through an inclusion, diversity, equity and access lens. J Acad Nutr Diet. 2023;123:247–252. 36116772 10.1016/j.jand.2022.09.014

[12] Schier HE, Linsenmeyer WR. Nutrition-related messages shared among the online transgender community: a netnography of YouTube vloggers. Transgend Health. 2019;4:340–349. 31872063 10.1089/trgh.2019.0048 PMC6918534

[13] Tricia K, Johnson MJ. Two case examples of reaching the hard-to-reach: low income minority and LGBT individuals. J Health Disparities Res Pract. 2017;10:11.

[14] Bonevski B, Randell M, Paul C, Reaching the hard-to-reach: a systematic review of strategies for improving health and medical research with socially disadvantaged groups. BMC Med Res Methodol. 2014;14:42. 24669751 10.1186/1471-2288-14-42 PMC3974746

[15] Creswell JW, Vicki L, Clark P. Designing and Conducting Mixed Methods Research. SAGE Publications, Inc; 2017.

[16] Fredriksen-Goldsen KI, Simoni JM, Kim HJ, The health equity promotion model: reconceptualization of lesbian, gay, bisexual, and transgender (LGBT) health disparities. Am J Orthopsychiatry. 2014;84:653–663. 25545433 10.1037/ort0000030 PMC4350932

[17] Bronfenbrenner U Environments in developmental perspective: theoretical and operational models. In: Friedman SL, Wachs TD, eds. Meas Environ Life Span Emerg Methods Concepts. American Psychological Association; 1999:3–28.

[18] Malterud K, Siersma VD, Guassora AD. Sample size in qualitative interview studies: guided by information power. Qual Health Res. 2016;26:1753–1760. 26613970 10.1177/1049732315617444

[19] Guillory J, Wiant KF, Farrelly M, Recruiting hard-to-reach populations for survey research: using Facebook and Instagram advertisements and inperson intercept in LGBT bars and nightclubs to recruit LGBT young adults. J Med Internet Res. 2018;20: e197. 29914861 10.2196/jmir.9461 PMC6028767

[20] Kallio H, Pietilä AM, Johnson M, Kangasniemi M. Systematic methodological review: developing a framework for a qualitative semi-structured interview guide. J Adv Nurs. 2016;72:2954–2965. 27221824 10.1111/jan.13031

[21] Buzzard IM, Faucett CL, Jeffery RW, Monitoring dietary change in a low-fat diet intervention study: advantages of using 24-hour dietary recalls vs food records. J Am Diet Assoc. 1996; 96:574–579. 8655904 10.1016/S0002-8223(96)00158-7

[22] Nathan S, Newman C, Lancaster K. Qualitative interviewing. In: Liamputtong P, ed. Handbook of Research Methods in Health Social Sciences. Springer; 2019:391–410.

[23] Oliver DG, Serovich JM, Mason TL. Constraints and opportunities with interview transcription: towards reflection in qualitative research. Soc Forces. 2005;84:1273–1289. 16534533 10.1353/sof.2006.0023 PMC1400594

[24] Braun V, Clarke V. One size fits all? What counts as quality practice in (reflexive) thematic analysis? Qual Res Psychol. 2021;18:328–352.

[25] Conway JM, Ingwersen LA, Moshfegh AJ. Accuracy of dietary recall using the USDA five-step multiple-pass method in men: an observational validation study. J Am Diet Assoc. 2004;104: 595–603. 15054345 10.1016/j.jada.2004.01.007

[26] Conway JM, Ingwersen LA, Vinyard BT, Moshfegh AJ. Effectiveness of the US Department of Agriculture 5-step multiple-pass method in assessing food intake in obese and nonobese women. Am J Clin Nutr. 2003;77:1171–1178. 12716668 10.1093/ajcn/77.5.1171

[27] Prochnik Estima CC, Da Costa RS, Sichieri R, Pereira RA, Da Veiga GV. Meal consumption patterns and anthropometric measurements in adolescents from a low socioeconomic neighborhood in the metropolitan area of Rio de Janeiro, Brazil. Appetite. 2009;52: 735–739. 19501773 10.1016/j.appet.2009.03.017

[28] Fulkerson JA, Telke S, Larson N, Berge J, Sherwood NE, Neumark-Sztainer D. A healthful home food environment: is it possible amidst household chaos and parental stress? Appetite. 2019;142: 104391. 31377322 10.1016/j.appet.2019.104391 PMC6779171

[29] Fulkerson JA, Story M, Neumark-Sztainer D, Rydell S. Family meals: perceptions of benefits and challenges among parents of 8to 10-year-old children. J Am Diet Assoc. 2008;108: 706–709. 18375230 10.1016/j.jada.2008.01.005

[30] Fairburn CG, Cooper Z, O’Connor M. Eating Disorder Examination. 17th ed. Oxford Press; 2014.

[31] Carter JC, Stewart DA, Fairburn CG. Eating disorder examination questionnaire: norms for young adolescent girls. Behav Res Ther. 2001;39:625–632. 11341255 10.1016/s0005-7967(00)00033-4

[32] Connell CL, Nord M, Lofton KL, Yadrick K. Food security of older children can be assessed using a standardized survey instrument. J Nutr. 2004;134:2566–2572. 15465749 10.1093/jn/134.10.2566

[33] National Academies of Sciences Engineering and Medicine. Measuring Sex, Gender Identity, and Sexual Orientation. The National Academies Press; 2022. 35286054

[34] Wardle J, Robb K, Johnson F. Assessing socioeconomic status in adolescents: the validity of a home affluence scale. J Epidemiol Community Health. 2002;56: 595–599. 12118050 10.1136/jech.56.8.595 PMC1732226

[35] Guglani L, Booza J, Havstad SL, Joseph CLM. Usefulness of a home affluence scale administered to urban adolescents with asthma to estimate the family’s socioeconomic status. Ann Epidemiol. 2015;25:855–860. 26303617 10.1016/j.annepidem.2015.07.010 PMC4641446

[36] US Department of Agriculture. US Department of Health and Human Services. Dietary Guidelines for Americans 2020–2025. 9th ed. US Government Publishing Office; 2020.

[37] Willett W Nutritional Epidemiology. Oxford Press; 2012.

[38] Shams-White MM, Pannucci TE, Lerman JL, Healthy eating index2020: review and update process to reflect the dietary guidelines for Americans, 2020–2025. J Acad Nutr Diet. 2023;123:1280–1288. 37201748 10.1016/j.jand.2023.05.015 PMC10524328

[39] Nagata JM, Compte EJ, Cattle CJ, Community norms for the eating disorder examination questionnaire (EDE-Q) among gender-expansive populations. J Eat Disord. 2020;8:74. 33292636 10.1186/s40337-020-00352-x PMC7722313

[40] Centers for Disease Control and Prevention. National Health and Nutrition Examination Survey (NHANES): Anthropometry Procedures Manual. Createspace Independent Publishing Platform; 2014.

[41] Centers for Disease Control and Prevention, National Center for Health Statistics. CDC growth charts: United States. http://www.cdc.gov/growth-charts/. Accessed November 1, 2024.

[42] Linsenmeyer W, Garwood S, Waters J. An examination of the sex-specific nature of nutrition assessment within the nutrition care process: considerations for nutrition and dietetics practitioners working with transgender and gender diverse clients. J Acad Nutr Diet. 2022;122:1081–1086. 35231663 10.1016/j.jand.2022.02.014

[43] Pitts SBJ, Jahns L, Wu Q, A non-invasive assessment of skin carotenoid status through reflection spectroscopy is a feasible, reliable and potentially valid measure of fruit and vegetable consumption in a diverse community sample. Public Health Nutr. 2018;21:1664–1670. 29455692 10.1017/S136898001700430X PMC6200334

[44] Whitehead RD, Mei Z, Mapango C, Jefferds MED. Methods and analyzers for hemoglobin measurement in clinical laboratories and field settings. Ann N Y Acad Sci. 2019;1450:147–171. 31162693 10.1111/nyas.14124 PMC6709845

[45] Daru J, Colman K, Stanworth SJ, De La, Salle B, Wood EM, Pasricha SR. Serum ferritin as an indicator of iron status: what do we need to know? Am J Clin Nutr. 2017;106:1634S–1639S. 29070560 10.3945/ajcn.117.155960 PMC5701723

[46] Okano SHP, Braga GC, Cantelli DAL, Filho LASP, Brito LGO, Lara LADS. Effect of testosterone formulations on hematocrit in transgender individuals: a systematic review [published online ahead of print July 16, 2024]. Andrology. doi:10.1111/andr.13695. 39011565

[47] Elin RJ, Wolff SM, Finch CA. Effect of induced fever on serum iron and ferritin concentrations in man. Blood. 1977;49:147–153. 830372

[48] Virginia B, Victoria C. Conceptual and design thinking for thematic analysis. Qualitative Psychology. 2022;9:3–26.

[49] Hayfield N, Huxley C. Insider and outsider perspectives: reflections on researcher identities in research with lesbian and bisexual women. Qual Res Psychol. 2015;12:91–106.

[50] Eliason MJ. Inside/out: challenges of conducting research in lesbian communities. J Lesbian Stud. 2016;20:136–156. 26701774 10.1080/10894160.2015.1061415

[51] Hellawell D Inside–out: analysis of the insider–outsider concept as a heuristic device to develop reflexivity in students doing qualitative research. Teach High Educ. 2006;11:483–494.

[52] Tang DTS. The research pendulum: multiple roles and responsibilities as a researcher. J Lesbian Stud. 2007;10: 11–27. 10.1300/J155v10n03_02 17210556

[53] Bridges D The ethics of outsider research. J Philos Educat. 2001;35: 371–386.

[54] Pitman GEV. Outsider/insider: the politics of shifting identities in the research process. Fem Psychol. 2002;12:282–288.

[55] Herman JL, Flores AR, O’neill KK. How Many Adults and Youth Identify as Transgender in the United States? The Williams Institute; 2022.

[56] Raifman S, DeVost MA, Digitale JC, Chen YH, Morris MD. Respondent-driven sampling: a sampling method for hard-to-reach populations and beyond. Curr Epidemiol Rep. 2022;91:38–47.

[57] Guest G, Namey E, Chen M. A simple method to assess and report thematic saturation in qualitative research. PLoS One. 2020;15:e0232076. 32369511 10.1371/journal.pone.0232076 PMC7200005

[58] Braun V, Clarke V. To saturate or not to saturate? Questioning data saturation as a useful concept for thematic analysis and sample-size rationales. Qual Res Sport Exerc Health. 2021;13:201–216.

[59] Kirkpatrick SI, Baranowski T, Subar AF, Tooze JA, Frongillo EA. Best practices for conducting and interpreting studies to validate self-report dietary assessment methods. J Acad Nutr Diet. 2019;119:1801–1816. 31521583 10.1016/j.jand.2019.06.010

[60] Basiotis PP, Welsh SO, Cronin FJ, Kelsay JL, Mertz W. Number of days of food intake records required to estimate individual and group nutrient intakes with defined confidence. J Nutr. 1987;117:1638–1641. 3655942 10.1093/jn/117.9.1638

[61] Baxter SD, Thompson WO, Davis HC, Johnson MH. Impact of gender, ethnicity, meal component, and time interval between eating and reporting on accuracy of fourth-graders’ self-reports of school lunch. J Am Diet Assoc. 1997;97:1293–1298. 9366868 10.1016/S0002-8223(97)00309-X

[62] Miles S, Scaife V. Optimistic bias and food. Nutr Res Rev. 2003;16:3–19. 19079933 10.1079/NRR200249

[63] Subar AF, Kirkpatrick SI, Mittl B, The automated self-administered 24-hour dietary recall (ASA24): a resource for researchers, clinicians, and educators from the National Cancer Institute. J Acad Nutr Diet. 2012;112:1134–1137. 22704899 10.1016/j.jand.2012.04.016 PMC3721511

[64] Bolland JE, Ward JY, Bolland TW. Improved accuracy of estimating food quantities up to 4 weeks after training. J Am Diet Assoc. 1990;90:1402–1404. 2212423

